# A “Short Isometric Construct” Reconstruction Technique for the Medial Collateral Ligament of the Knee

**DOI:** 10.1016/j.eats.2022.10.005

**Published:** 2023-01-18

**Authors:** Kyle A. Borque, Simon Ball, Ethan Sij, Andrew A. Amis, Mitzi S. Laughlin, Mary Jones, Andy Williams

**Affiliations:** a Houston Methodist Hospital, Houston, Texas, U.S.A.; b McGovern Medical School, UT Health, Houston, Texas, U.S.A.; c Houston Methodist Research Institute, Houston, Texas, U.S.A.; d the Fortius Clinic, FIFA Medical Centre of Excellence, London, United Kingdom; e the Imperial College London, London, United Kingdom

## Abstract

Recently there has been increased focus on the medial collateral ligament (MCL) and the role the medial ligament complex plays in preventing valgus and external rotation, especially in the setting of a combined ligament injury. Multiple surgical techniques purport to reproduce “normal anatomy”; however, only one technique addresses the deep MCL fibers and the prevention of external rotation. Thus we describe the “short isometric construct” MCL reconstruction which is stiffer than the anatomic reconstructions. The “short isometric construct” technique resists valgus throughout range of motion whereas its obliquity resists tibial external rotation, helping to reduce the risk of anterior cruciate ligament graft re-rupture.

There is renewed focus on the medial ligament complex, especially in the setting of a combined ligament injury.^1^, ^2^, ^3^ Willinger et al.^4^ reported that 67% of presumed “isolated anterior cruciate ligament (ACL)” injuries actually had injury to the medial ligament complex. The importance of identifying and treating MCL lesions has been highlighted in recent studies showing increased ACL graft failure with unaddressed MCL laxity.^5^, ^6^, ^7^

The medial ligament complex comprises: the superficial MCL (sMCL), the deep MCL (dMCL), and the posterior oblique ligament (POL).^8^^,^^9^ The sMCL is the primary restraint to valgus stress, and the POL resists internal rotation when the knee is close to full extension.^8^^,^^9^ Contrary to previous dogma, external rotation is resisted principally by the dMCL in the functional weightbearing range near knee extension, then by 30° it shares the load with the sMCL, which predominates only in deeper flexion angles. Hence, anteromedial rotatory instability is an ACL plus dMCL problem.^2^

Multiple surgical techniques purport to reproduce “normal anatomy” even though there is debate about the MCL bony attachments.^10^, ^11^, ^12^, ^13^ Several groups have recently described techniques that address the external rotation role of the dMCL.^14^, ^15^, ^16^ The authors prefer a short isometric reconstruction construct because it is stiffer and resists valgus and axial rotation. The philosophy is always to undertake suturing first to restore native MCL structures and then protect them while healing with a reconstruction that has to be biomechanically true and therefore effective in protecting soft tissue repairs, but not necessarily “anatomic.”

## Surgical Technique

Indications for MCL reconstruction are medial joint opening on valgus stress to grade 3 at 30° flexion in athletes or any degree with the knee in full extension in all patients; symptomatic non-acute grade 2 MCL laxity at 30°, a positive dial test result when the increased external rotation is due to anteromedial rotatory instability; in the presence of an ACL rupture, concomitant grade 2 or 3 MCL laxity or a positive Slocum test result^10^ (failure of external rotation to abolish a positive anterior drawer); and cases of sMCL avulsion from the tibia with the sMCL lying in the joint or superficial to the pes anserine tendons.

Preoperative evaluation includes standard radiography and magnetic resonance imaging. The magnetic resonance imaging scan should be evaluated to determine not only the presence of injury, but also the specific location of injury to the MCL complex. The patient is positioned supine, and an examination with the patient under anesthesia is performed and compared to the uninjured knee. When assessing valgus laxity, it is critical to do so with the tibia reduced on the femur and application of compressive force across the knee joint.^1^ An easy error in the ACL-deficient knee is to use the examination method of holding the leg at the proximal tibia with the patient’s foot/ankle in the examiner’s axilla. This allows anterior tibial translation because of the effect of gravity on the femur. In such a subluxed knee, a “false negative” can result because the subluxation takes up slack in the MCL.

**Fig 1:**
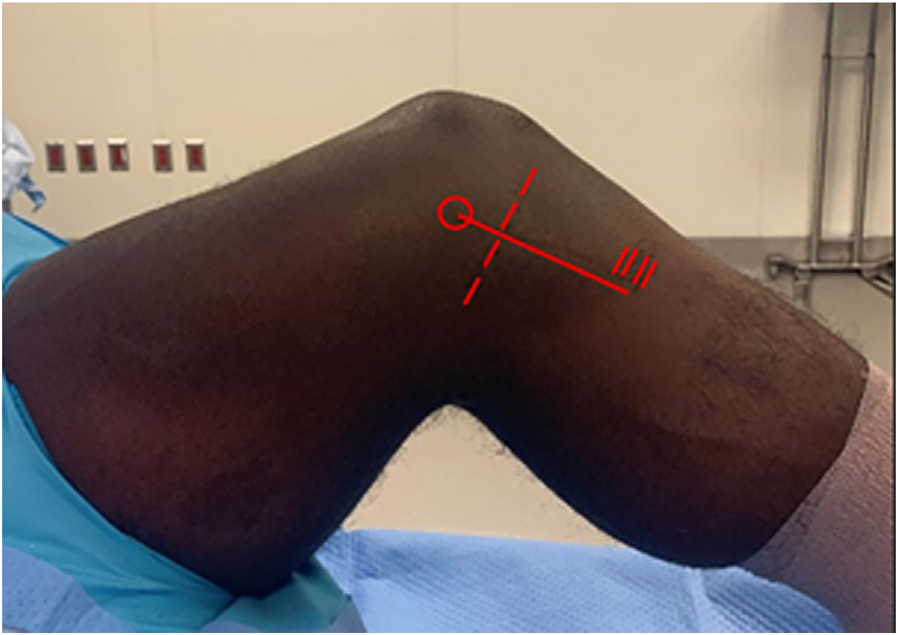
Medial view of a left knee identifying landmarks for the short isometric construct medial collateral ligament reconstruction: the medial femoral epicondyle (red circle), the medial joint line (red dotted line), the pes anserine insertion (2 parallel red lines), and the incision (straight line from medial femoral condyle to pes anserine insertion).

In contrast this examination technique is ideal in the posterior cruciate ligament (PCL)–deficient knee because gravity reduces the knee. The limb is positioned with a footrest and a side support. A pneumatic tourniquet is applied and inflated. Landmarks including the medial epicondyle, the joint line, and the pes anserine insertion are marked (Fig 1). Diagnostic arthroscopy is performed, making note of the location of medial joint opening in relation to the medial meniscus. Joint opening below the meniscus is suggestive of a distal MCL injury, whereas opening above the meniscus suggests a proximal injury, although the injury may involve both proximal and distal portions of the MCL.

With the knee in 45° to 60° of flexion, an incision is made along the line of the sMCL from the medial epicondyle to posterior to the insertion of the pes anserine (Video). Sharp dissection is carried down to layer 1, the deep fascia. Care is taken to identify the infrapatellar branch of the saphenous nerve and protect it. Layer 1 is then sharply incised in line, with its fibers revealing layer 2, the sMCL. Suture repairs or plication of the dMCL and sMCL are then performed, according to location and chronicity.^1^

**Fig 2:**
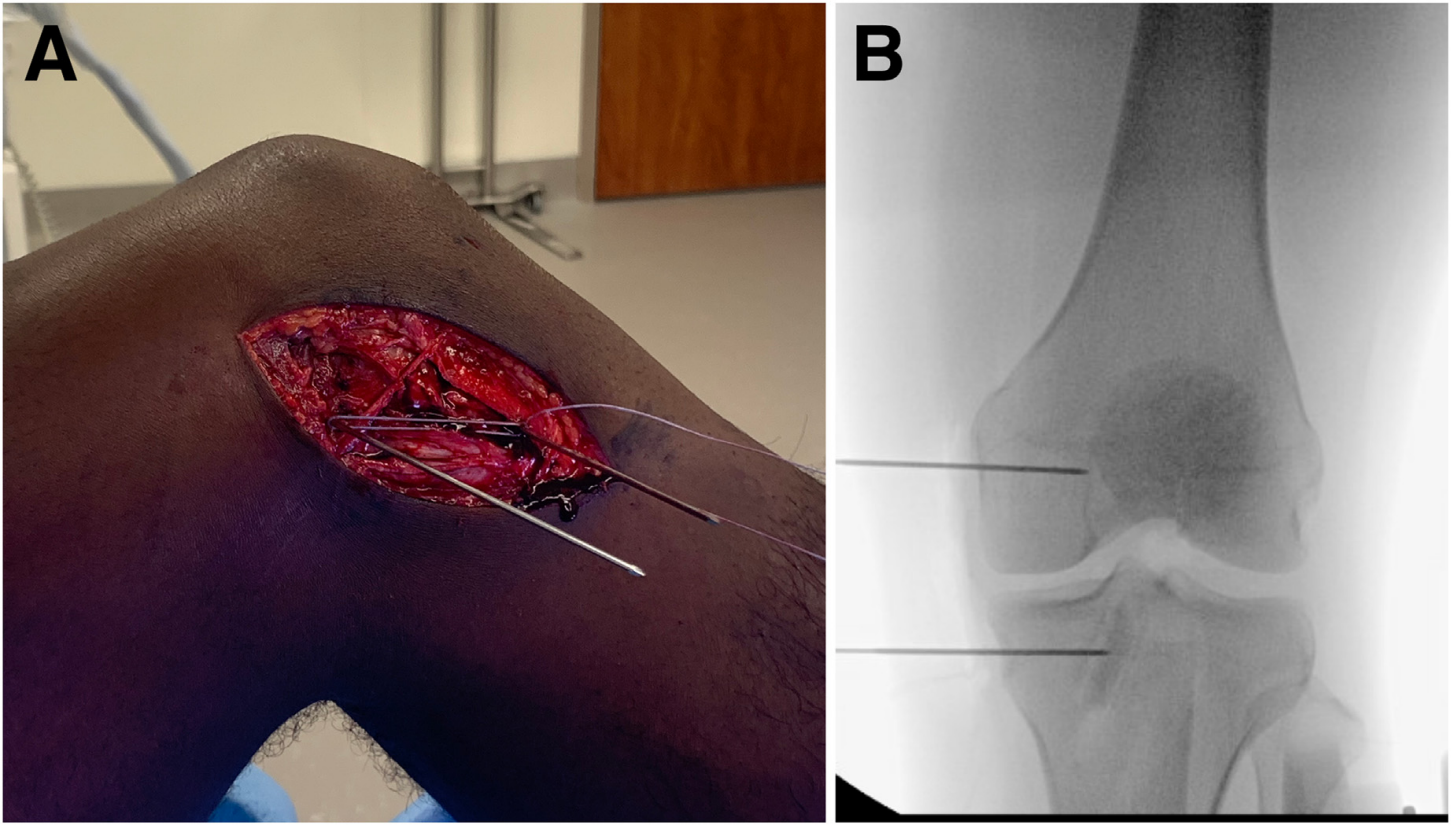
(A) Medial view of a left knee with a suture tied around 2 K-wires placed at the femoral and tibial insertions of the short isometric construct for medial collateral ligament reconstruction. (B) Anteroposterior fluoroscopic image confirming correct placement of the K-wires.

**Fig 3:**
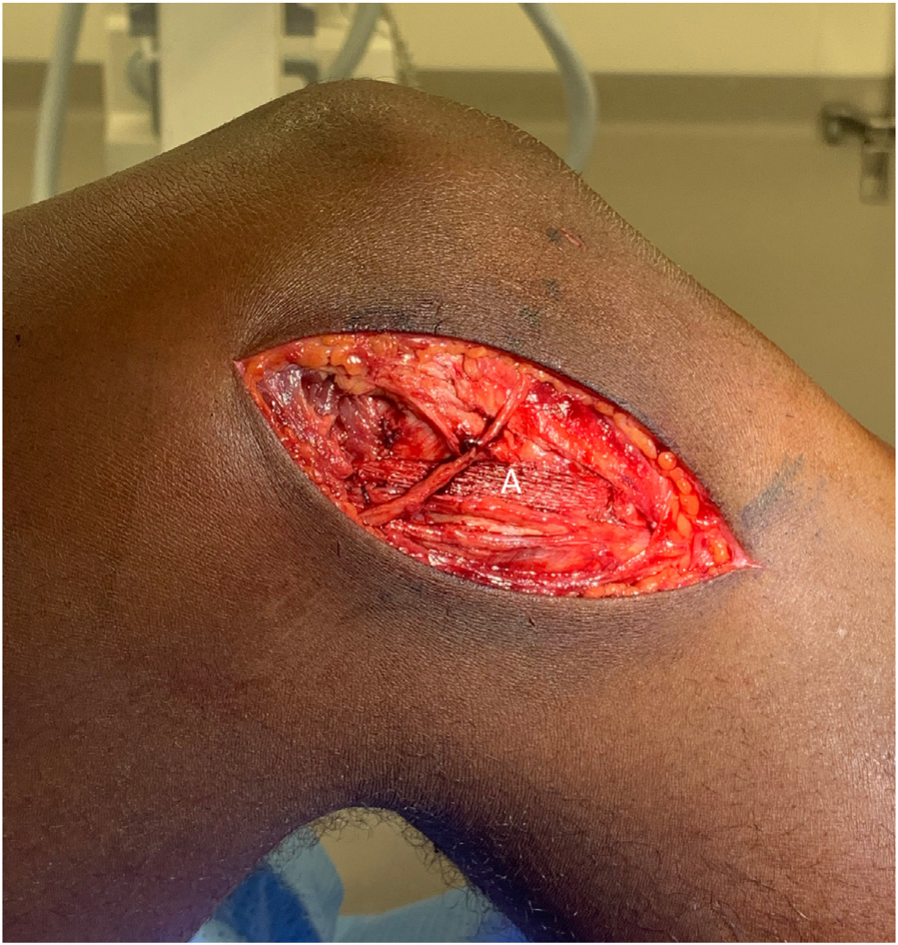
Medial view of the left knee showing a completed MCL reconstruction with a polyester tape passed underneath an infrapatellar branch of the saphenous nerve and fixed in the tibia and femur with interference screws.

A short isometric MCL reconstruction is performed to protect the MCL repairs during healing. Two Kirschner wires (K-wires) are placed under fluoroscopic control: at the center of the medial epicondyle, which is the most isometric point,^9^ and at a point 15 to 20 mm below the tibial articular margin just anterior to the halfway point between the anterior and posterior margins of the sMCL (Fig 2). In extension, this construct has an obliquity that allows it to resist tibial external rotation. Isometry of the construct is critical and is assessed with a suture passed around both of the K-wires while the knee is taken through a full range of flexion/extension while maintaining “neutral” axial rotation (determined by examining the contralateral leg before surgery) (Fig 3). A nonisometric construct results in loss of efficacy and restriction of flexion or extension. Nonisometric K-wire placement is corrected in most cases by adjustment of the position of the femoral K-wire. Tightening in flexion requires a more posterior femoral position, and tightness in extension a more anterior one.

Once isometry has been confirmed, 6.5mm sockets are drilled at the location of each K-wire. A 7 mm polyester tape (Infinity Lock, Xiros, Leeds UK) is fixed first at the femoral insertion with a 7x20mm interference screw (QuickStart; Innovate Orthopaedics, Huddersfield, United Kingdom). The tape is then passed deep to the infrapatellar branch of the saphenous nerve, and with the knee at 30° flexion, neutral tibial rotation (based on pre-operative examination of the contralateral knee) and a varus force applied, fixed at the tibial insertion with a second interference screw (Fig 3). Radiography is used to confirm implant placement.

**Fig 4:**
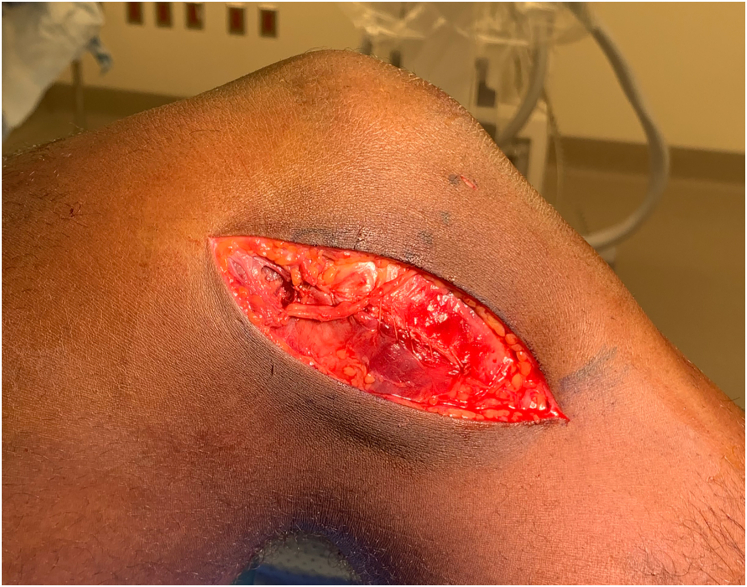
Medial view of the left knee with layer 1 closed over the medial collateral ligament reconstruction and deep to a branch of the saphenous nerve.

The knee is then brought through a full range of motion, and graft isometry is reconfirmed. Layer 1 is then double-breasted over the reconstruction (Fig 4). The wound is closed in layers, and a compressive dressing is applied.

## Postoperative Rehabilitation Protocol

This protocol is used by the senior surgical author (A.W.). Full active and passive extension should be achieved as soon as possible. The exception is in the unusual scenario of a POL injury that required repair, when extension is limited to 0° without early hyperextension. Flexion should progress without restriction unless concomitant PCL surgery requires it. Isometric quadriceps contractions are begun immediately after surgery.

The knee is placed in a hinged knee brace for 6 weeks after surgery while mobilizing and in bed for the first 2 weeks. The brace is removed for icing, exercises, and comfort at rest. The patient is kept toe touch weightbearing for the first 2 weeks, followed by partial weightbearing (50% body weight) for 2 weeks, then full weightbearing. For the first 6 weeks, the patient is instructed to walk with the foot directed straight forward, rather than the natural 10° of external rotation, in an effort to offload repairs and reconstruction. Closed chain quads exercises commence at the start of the third week. At 6 weeks, the patient is transitioned out of the brace and rehabilitation is unrestricted focusing on strength of the limb and “core,” range of motion, and proprioception.

## Discussion

The importance of identifying and addressing MCL laxities especially in the setting of ACL reconstruction has been emphasized recently.^3^ Ahn et al.^7^ showed a 10-fold increase in re-rupture rate after primary ACL reconstruction in the setting of a grade I MCL injury and a 13-fold increase with a grade II MCL injury. These findings are supported by the Swedish Ligament Registry where Svantesson et al.^5^ showed increased risk of ACL re-rupture with nonoperative treatment of MCL injuries, but this risk was eliminated if the MCL was addressed surgically. In addition, Alm et al.^6^ have shown the importance of addressing medial laxity in the setting of revision ACL reconstruction, where preoperative medial instability increased risk of re-rupture 17-fold.

Based on recent anatomic and biomechanical studies,^2^^,^^3^^,^^8^^,^^17^ it seems the role of the POL has hitherto been overemphasized. Only rarely does the POL need reconstructing; in the uncommon scenario of posteromedial rotatory instability with a combined PCL injury, simple repair is usually enough in other scenarios. The authors prefer a short reconstruction construct with a slight obliquity because it has less potential to stretch when compared to other techniques with a long sMCL component and no dMCL.^11^^,^^18^^,^^19^ Other reconstruction techniques have been described recently, the majority using 2 or 3 limbs to reconstruct the medial ligament complex. The benefit of this technique is that one limb is used to restore both valgus and rotational stability.^20^^,^^21^ Being isometric, the construct resists valgus throughout range of motion whereas its obliquity resists tibial external rotation.

The choice of synthetic graft is based on our experience that soft tissue grafts tend to stretch when placed on the medial side of the knee—likely because of cyclic loading. Synthetic grafts’ stiffness resists creep, and we have not seen the problems attributed to synthetic grafts in an intra-articular (cruciate ligament) setting when used in the extra-articular soft tissue envelope.

The MCL reconstruction is always performed in combination with a primary repair of the tissues.^1^ The repair technique varies based on the location and chronicity of the MCL injury, but, by combining a repair and a reconstruction, the reconstruction serves only to protect the repair while it is healing. Once the natural MCL tissue becomes functional, the reconstruction is redundant. Pearls and pitfalls and advantages and disadvantages of our technique are described in Tables 1 and 2, respectively.

**Table 1:** Pearls and Pitfalls

Pearls
The synthetic ligament creates a high strength construct with minimal creep, preventing the MCL from stretching out over time.
Valgus and rotational stability can be restored with one construct
Use fluoroscopy to confirm wire placement before drilling sockets
Pitfalls
It is vital to place the construct in an isometric position or it can constrain motion.
The femoral socket location can conflict with a PCL femoral tunnel, though this risk is decreased in this technique, as sockets are used instead of tunnels.
In skeletally immature patients, the construct must be placed in the epiphysis of both the femur and tibia to avoid tethering across the physis.

MCL, medial collateral ligament.

**Table 2:** Advantages and Disadvantages

Advantages
Isometric construct resists valgus throughout range of motion
Obliquity resists tibial external rotation
Stiffer than anatomic reconstructions
Disadvantages
A nonisometric construct results in loss of efficacy and range of motion
In skeletally immature patients, the construct must be placed in the epiphysis of both the femur and tibia to avoid tethering across the physis.

## Supplementary Data

ICMJE author disclosure forms

Video In this Technical Note, we demonstrate a “short isometric construct” reconstruction technique for the medial collateral ligament of the knee. This technique aims to restore native medial collateral ligament structures with a repair and then protect them while healing with the “short isometric construct” reconstruction.
